# JAK Inhibitor Upadacitinib Induces Remission in Refractory Immune‐Related Colitis Triggered by CTLA‐4 and PD‐1 Inhibitor Combination Therapy in Malignant Pleural Mesothelioma: A Case Report

**DOI:** 10.1002/cnr2.70032

**Published:** 2024-10-28

**Authors:** Masashi Kono, Yoriaki Komeda, Hisato Kawakami, Satoru Hagiwara, George Tribonias, Kohei Handa, Shunsuke Omoto, Mamoru Takenaka, Hiroshi Kashida, Naoko Tsuji, Masatoshi Kudo

**Affiliations:** ^1^ Department of Gastroenterology and Hepatology Kindai University Faculty of Medicine Osaka Japan; ^2^ Department of Medical Oncology Kindai University Faculty of Medicine Osaka Japan; ^3^ Department of Gastroenterology Red Cross Hospital Athens Greece

**Keywords:** CTLA‐4 inhibitor, irAE, JAK inhibitor, PD‐1 inhibitor, upadacitinib

## Abstract

**Background:**

Immune checkpoint inhibitors have demonstrated efficacy against various cancers; however, there is a rising incidence of immune‐related colitis. Some cases of immune‐related colitis prove resistant to treatment, even with the administration of glucocorticoids or infliximab, and there is currently no established standard treatment for such cases.

**Case:**

The patient, a 73‐year‐old male, had undergone combination therapy for malignant pleural mesothelioma for 2 years, utilizing both ipilimumab (a CTLA‐4 inhibitor) and nivolumab (a PD‐1 inhibitor). Unfortunately, the treatment led to side effects, specifically immune‐related adverse event (irAE) enterocolitis. Steroid and infliximab treatment failed to improve the patient's condition. Treatment with tacrolimus was attempted, but the patient remained unresponsive. Subsequently, 45 mg of upadacitinib, a Janus kinase (JAK) inhibitor, was administered. Symptoms improved rapidly following upadacitinib administration, and endoscopy also revealed positive results. With the increasing incidence of immune‐related colitis, some patients have become resistant to treatment with glucocorticoids and infliximab. In this case, the irAE enterocolitis was improved by upadacitinib administration.

**Conclusion:**

In cases where immune‐related colitis proves resistant to treatment with glucocorticoids, infliximab, or tacrolimus, upadacitinib represents a potential option as a JAK inhibitor.

## 1 Introduction

Immune checkpoint inhibitors (ICIs) have been reported to be effective against various cancers. Their use has increased in recent years, and the incidence of immune‐related colitis has increased accordingly. Immune‐related colitis is a complication that causes the highest number of hospitalizations, treatment discontinuations, and deaths among patients receiving ICIs [1]. Recently, inhibitors of the Janus kinase (JAK)‐signaling and transcription activator (STAT) pathway, particularly tofacitinib, have been reported in ICI colitis, showing clear clinical remission of colitis [2].

As with ulcerative colitis, current treatment guidelines for immune‐mediated colitis recommend discontinuing ICIs and antidiarrheal drugs.

Loperamide hydrochloride can be used with dietary restrictions when symptoms are relatively mild. However, if colitis is difficult to control, glucocorticoids are recommended, followed by biologic agents such as infliximab [3, 4, 5].

However, some patients are resistant to these biological agents and, in such cases, there is no clear standard treatment. In this case, a patient with immune‐related colitis resistant to biological agents achieved remission through the administration of the JAK inhibitor upadacitinib [6], establishing it as a novel and effective treatment strategy.

## 2 Case

The patient was a 73‐year‐old man who presented with bilateral pleural effusions in June 2021. He was diagnosed with malignant pleural mesothelioma in August 2021 and had been treated for 2 years with the CTLA‐4 inhibitor ipilimumab and the PD‐1 inhibitor nivolumab for malignant pleural mesothelioma. His medical history was unremarkable, and he had no history of smoking or drinking.

In March 2023, he experienced watery diarrhea more than 10 times a day and developed immune‐related adverse event (irAE) enterocolitis.

The frequency of his diarrhea increased to seven or more times a day compared with baseline, and fecal incontinence was also observed. This led to limitations in his activities of daily living, and he was diagnosed with CTCAE grade 3 [7, 8].

Despite receiving treatment with steroids (60 mg/day) and infliximab (5 mg/kg), his condition did not improve; consequently, he was transferred to Kindai University Hospital for advanced therapy. At the time of transfer, the patient was taking alfacalcidol for osteoporosis due to high doses of steroids and sulfamethoxazole–trimethoprim for the prevention of opportunistic infections such as fungal diseases.

**FIGURE 1:**
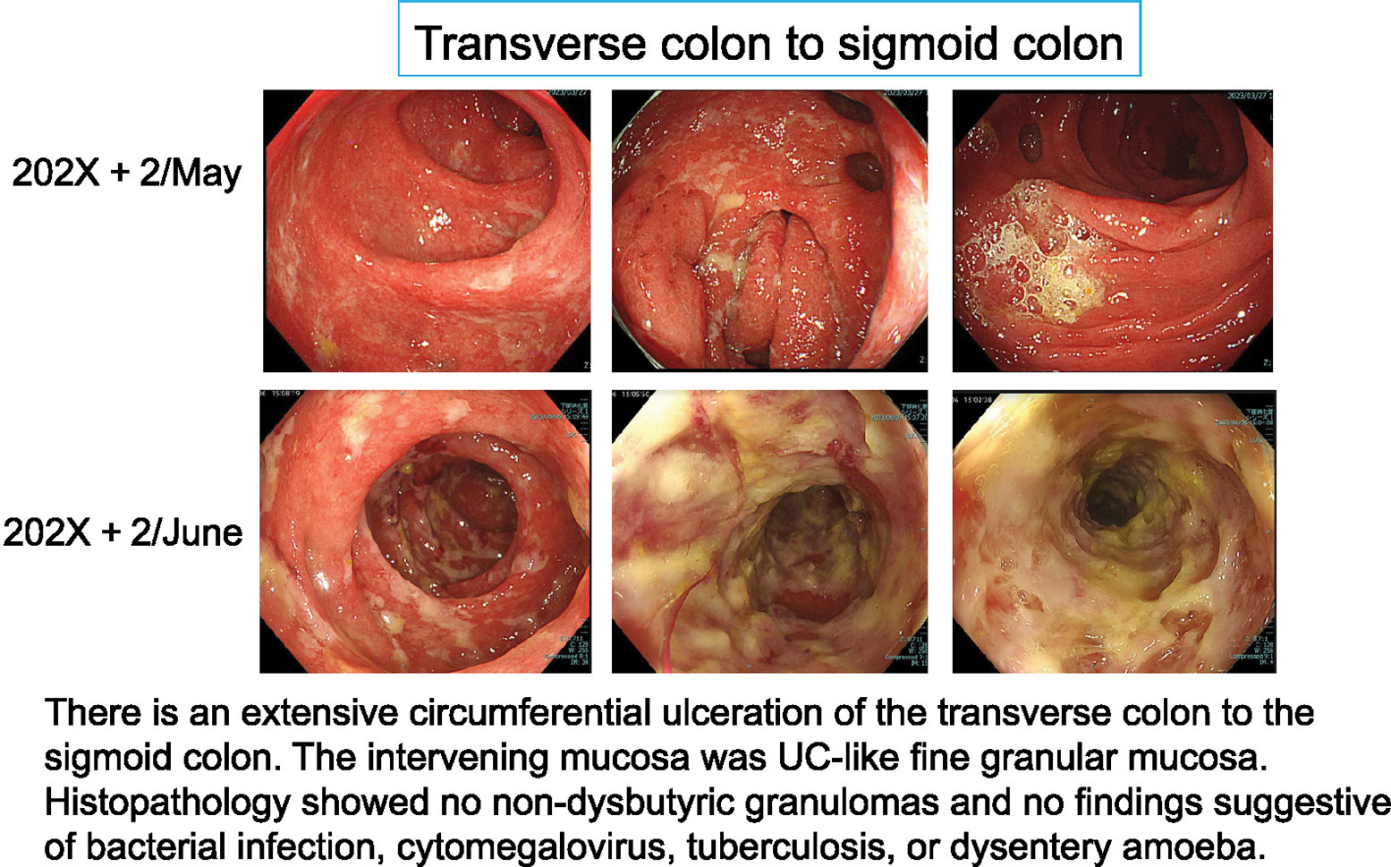
Colonoscopy image from the previous hospital.

**FIGURE 2:**
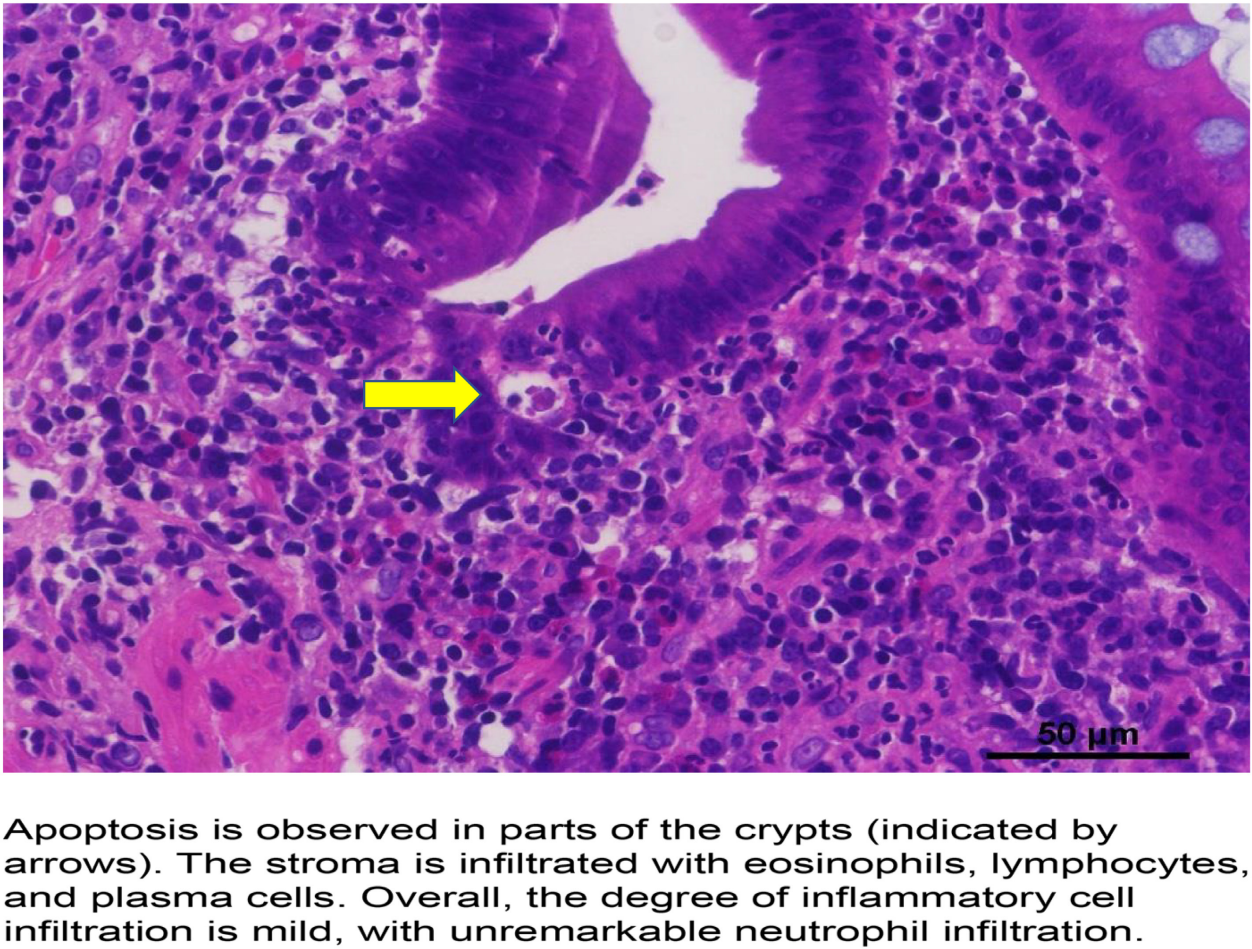
Histopathological examination of the colonic mucosa. H&E ×40 magnification.

A colonoscopy performed by a previous physician revealed widespread ulcers circumferentially from the transverse colon to the sigmoid colon, and the intervening mucosa was fine granular mucosa with diffuse inflammation throughout the colon, similar to moderate UC (Figure 1). The patient was diagnosed as high‐risk according to the risk‐based irAE enterocolitis endoscopic initial classification [9]. Histopathological examination revealed no noncaseating granulomas but decreased mucus production in crypts, cryptitis, and apoptosis in epithelial cells. CD4+ and CD8+ lymphocytes were detected (Figure 2).

A colonoscopy was performed to assess the treatment efficacy during steroid therapy, which was then being reduced from 60 to 40 mg/day. The colonic mucosal biopsy revealed that the patient also had CMV enteritis and pseudomembranous enteritis. Consequently, anti‐CMV drugs and metronidazole were administered. While CMV enteritis and pseudomembranous enteritis were being treated, CAP therapy was expected to improve irAE enteritis itself; however, no improvement was observed.

**FIGURE 3:**
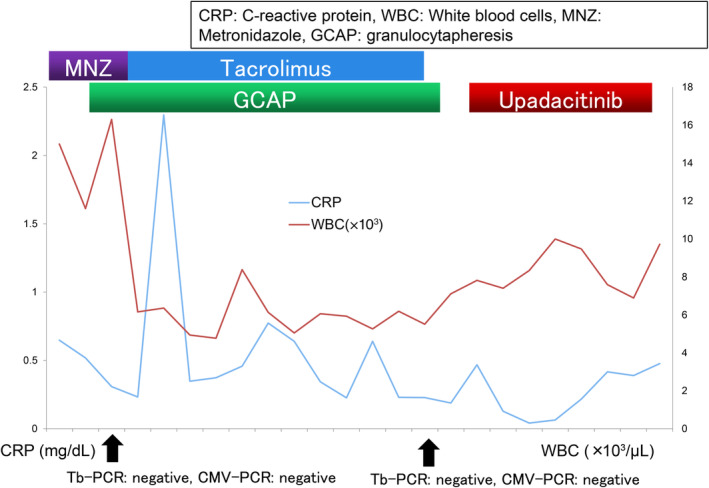
Progress of drug treatment.

Two weeks of treatment with tacrolimus (2–4 mg adjusted for trough values: 10–20 ng/mL) was attempted after waiting for improvement of the CMV enteritis and pseudomembranous enteritis; however, the patient was unresponsive. Therefore, 45 mg of the JAK inhibitor upadacitinib was administered. After upadacitinib administration, the frequency of defecation rapidly improved from more than 10 to less than 3 in a few days, and the blood samples showed no inflammation (Figure 3).

**FIGURE 4:**
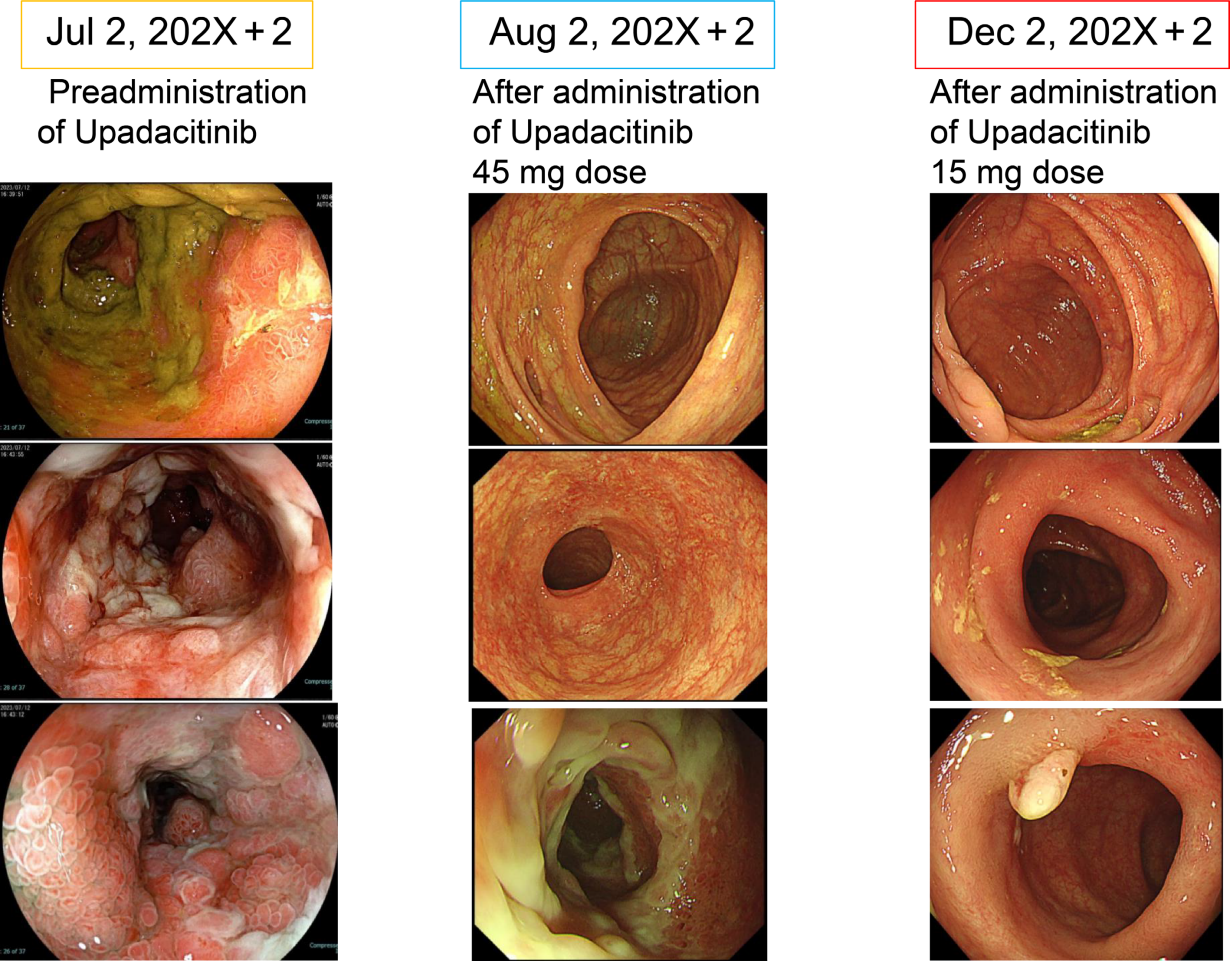
Colonoscopy images before and after UPA administration.

Although the upadacitinib dose was reduced to 15 mg after 8 weeks of the first dose, there was no recurrence, and endoscopy showed improvement (Figure 4). Eventually, mucosal remission, that is, complete disappearance of inflammation and ulcers in the colon mucosa confirmed by endoscopy, was achieved after about 6 months, leading to the discontinuation of upadacitinib. There have been no recurrences in the 6 months since the discontinuation of upadacitinib. There has also been no progression of malignant pleural mesothelioma, and the patient continues to be monitored.

## 3 Discussion

ICIs have been reported as effective against various cancers; consequently, their usage and the incidence of immune‐related colitis are on the rise.

Regarding irAE enterocolitis due to ICI administration, the frequency of enteritis complications has been reported at 18%–23% when anti‐CTLA‐4 antibodies and anti‐PD‐1 inhibitors are used in combination [10]; this tends to be higher than when antibodies are used alone.

Nevertheless, there is a probability that some cases may exhibit resistance to glucocorticoids and biological agents, posing a challenge in treatment. Vedolizumab, although considered a third‐line drug in such cases [11], was not administered in this case as the symptoms were severe.

In patients with severe UC, vedolizumab may require some time to show its effects. Therefore, it is not recommended when rapid improvement is crucial.

A previous study [12] reported the effectiveness of tacrolimus in cases of irAE enteritis resistant to treatment with steroids and influenza; however, the patient did not respond.

JAK inhibitors have become an important treatment modality for managing inflammatory bowel disease (IBD). Despite the similarities with ICI colitis, the etiology of IBD has differences, including a self‐perpetuating inflammatory process characterized by a relapsing–remitting course independent of previous patient treatment [13, 14].

However, these two disease states share extensive molecular features regarding the inflammatory pathways involved and frequently exhibit overlapping clinical and endoscopic manifestations [15, 16]. Although tofacitinib has been reported, the clinical use of other JAK inhibitors for the treatment of irAE enteritis is currently limited [17]. The efficacy of IL12/23 inhibitors has also been reported [18]. However, a clear treatment method has yet to be established. Previous studies have shown that JAK inhibitors, such as upadacitinib, are effective in treating IBD. However, there have been limited reports on JAK inhibitors for irAEs, and this study is one of the first to demonstrate their effectiveness. Other options for treatment, such as vedolizumab and tacrolimus, are available as third‐line drugs, but they may take some time to show their effects. In this regard, upadacitinib is expected to have a rapid effect, enabling a rapid response in severe cases [6, 11, 12].

Considering the convenience of oral administration and the rapid onset of action, upadacitinib may be an effective treatment option for patients with irAE enterocolitis. We believe that further verification is necessary for future studies, such as prospective trials.

We observed a marked improvement in irAE enterocolitis with the administration of upadacitinib; therefore, considering this occurrence as extremely rare, we report it here. As the development of malignant tumors is a known side effect of JAK inhibitors, we performed colonoscopy, which revealed mucosal remission. Subsequently, the medication was discontinued, and a follow‐up was conducted.

This study demonstrated the efficacy of upadacitinib as a treatment option for patients with immune‐related enterocolitis resistant to steroids and infliximab. Rapid symptom improvement was observed with upadacitinib, and complete mucosal remission was confirmed by endoscopy. Therefore, upadacitinib is considered a promising treatment option for patients with irAEs that do not respond to conventional therapies.

Future studies should include prospective trials to evaluate the long‐term safety and efficacy of upadacitinib. Additionally, further validation is required to expand the range of indications for upadacitinib.

This case is reported as an example of a new therapeutic approach for severe irAEs. The use of JAK inhibitors has provided new hope for patients with difficult‐to‐treat immune‐related enterocolitis.

## Author Contributions

**Masashi Kono:** conceptualization, formal analysis, investigation, methodology, project administration, writing – original draft. **Yoriaki Komeda:** conceptualization, formal analysis, investigation, methodology, project administration, writing – original draft. **Hisato Kawakami:** supervision. **Satoru Hagiwara:** data curation. **George Tribonias:** data curation. **Kohei Handa:** data curation. **Shunsuke Omoto:** data curation. **Mamoru Takenaka:** data curation. **Hiroshi Kashida:** data curation. **Naoko Tsuji:** data curation. **Masatoshi Kudo:** writing – review and editing.

## Ethics Statement

This study was approved by the Ethics Committee of Kindai University Hospital (No. RR05‐27).

## Consent

The patient provided written informed consent for the publication of this case report and accompanying images.

## Conflicts of Interest

The authors declare no conflicts of interest.

## Data Availability

Data sharing not applicable to this article as no datasets were generated or analyzed during the current study.
